# Enhanced Protective Coatings Based on Nanoparticle fullerene C60 for Oil & Gas Pipeline Corrosion Mitigation

**DOI:** 10.3390/nano9101476

**Published:** 2019-10-17

**Authors:** Xingyu Wang, Fujian Tang, Xiaoning Qi, Zhibin Lin, Dante Battocchi, Xi Chen

**Affiliations:** 1 Department of Civil and Environmental Engineering, North Dakota State University, Fargo, ND 58018, USA; Xingyu.wang@ndsu.edu; 2 State Key Laboratory of Coastal and Offshore Engineering, School of Civil Engineering, Dalian University of Technology, Dalian 116024, China; ftang@dlut.edu.cn; 3 Department of Coatings and Polymeric Materials, North Dakota State University, Fargo, ND 58018, USA; xiaoning.qi@ndsu.edu (X.Q.); bante.battocchi@ndsu.edu (D.B.); 4 Guilin University of Technology, Guilin Guangxi 541004, China; xi.chen_01@outlook.com

**Keywords:** nano-modified high-performance coating, dispersion methods, fullerene-C60, corrosion mitigation, nanocomposite, gas and oil pipelines

## Abstract

Corrosion accounts for huge maintenance cost in the pipeline community. Promotion of protective coatings used for oil/gas pipeline corrosion control, in terms of high corrosion resistance as well as high damage tolerance, are still in high demand. This study was to explore the inclusion of nanoparticle fullerene-C60 in protective coatings for oil/gas pipeline corrosion control and mitigation. Fullerene-C60/epoxy nanocomposite coatings were fabricated using a solvent-free dispersion method through high-speed disk (HSD) and ultrasonication. The morphology of fullerene-C60 particles was characterized by transmission electron microscopy (TEM), and dynamic light scattering (DLS). The data analysis indicated that the nanoparticles were effectively dispersed in the matrix. The performance of the nanocomposites was investigated through their mechanical and electrochemical properties, including corrosion potential, tensile strength, strain at failure, adhesion to substrate, and durability performance. Dogbone shaped samples were fabricated to study the tensile properties of the nanocomposites, and improvement of strength, ultimate strain, and Young’s modulus were observed in the C60/epoxy specimens. The results demonstrated that the C60/epoxy composite coatings also had improvements in adhesion strength, suggesting that they could provide high damage tolerance of coatings for engineering applications. Moreover, the electrochemical impedance spectroscopy (EIS) results generated from the accelerated durability test revealed that the developed fullerene-C60 loaded composite coatings exhibited significantly improved corrosion resistance. The nanocomposite with 0.5 and 1.0 wt.% of C60 particles behaved as an intact layer for corrosion protection, even after 200-h salt spray exposure, as compared to the control coating without nanofiller in which severe damage by over 50% reduction was observed.

## 1. Introduction

Corrosion has been a leading cause of metallic oil/gas pipeline failures in the United States and worldwide [1,2,3,4]. A report revealed that there are over 2.6 million miles of gas and oil pipeline in United States. Most of the pipelines are fabricated by low-carbon steel, and severe corrosion failures can be developed when they are exposed to corrosive media during transporting oil and gas [5]. As of 2014, the reported cost of corrosion in the oil and gas industry was more than $17 billion for the United States [6]. Despite the fact that significant efforts have been targeted in corrosion control and mitigation, there are still challenges to develop effective corrosion prevention techniques [7,8,9,10,11].

In the last two decades, carbon-based nanoparticles have attracted great attention due to their ability to provide outstanding mechanical, tribological, and electrical properties with the various dimensions and geometrical shapes [12,13,14,15,16,17,18,19]. One particularly promising application of carbon-based nanoparticles is to assemble high-performance nanocomposites by incorporating nanofiller reinforcement into polymers [13] and coatings [18,19,20]. The enhancements of carbon-based nanocomposites are often considered to be associated with the shape and size of the nanofillers [14,15]. Nanofillers can be defined as zero, one, or two-dimensional materials [16]. Fullerene-C60 is a typical 0-dimensional spherical nanofiller that contains only carbon atoms, consisting of 12 pentagons and 20 hexagons arranged in a cage-like structure [17]. Their unique shapes provide a different combination of properties and assist the polymetric coating in overcoming their limitations [19,21,22,23,24,25,26,27]. In particular, fullerene-C60/polymer nanocomposites demonstrate enhanced mechanical and anti-corrosion properties, as compared to neat polymer materials [28,29]. 

Fullerene-C60 particles have been investigated as a nanofiller in polymer reinforcement due to their unique aromatic character and nanosized diameter [30]. Zuev et al. [29] suggested strong reinforcement could be obtained with a small amount of fullerene nanofillers in epoxy matrix, which should be less than 0.5 wt.%. Otherwise, agglomerates will be created with the excess amount of C60 nanoparticles, which leads to degradation on coating performance. Pikhurow et al. [28] reported that the addition of fullerene-C60 particles improved the Young’s modulus and tensile strength of epoxy resin. The maximum reinforcement was observed in the sample containing 0.08 wt.% of fullerene-C60 particles. Differently, other authors [30] mentioned that the maximum reinforcement on mechanical properties and elastic resilience was observed with the addition of 2 wt.% of C60 in poly(styrene-b-butadiene-b-styrene (SBS)), and the tensile strength increased almost 13 times compared with base polymer. Meanwhile, Ogasawara et al. [31] also pointed out that the greatest improvement was obtained with 1.0 wt.% of fullerene-C60 nanoparticles in the epoxy, with both increased tensile strength and strain. In addition, compared with neat epoxy, researchers [17] suggested both enhanced anti-corrosion and tribological properties were observed after the incorporation of C60 particles. C60/epoxy nanocomposites containing C60 particles from 0.25 to 1.0 wt.% were fabricated. However, the performance of the coatings was weakened when the content of C60 was higher than 0.5 wt.%; hence, this observation may result from large number of aggregated nanoparticles. Clearly, fullerene-C60 nanoparticle showed its potential to the development of high-performance coatings, but the current findings as observed in the literature is still unclear, as there were still in high variances in many academic studies. Specifically, one may concern appropriate content of nanoparticles as demanded for a coating system; however, the suggestions of fullerene-C60 content in coating system were sources of conflict among different researchers. Moreover, most of existing studies on fullerene-C60 particles as nanofillers in polymer system have been directed to mechanical properties of the nanocomposites [28,29,31]. As compared, few studies [17] discussed corrosion protection behaviors of fullerene-C60 particle reinforced coatings, which are critical properties as required for metallic pipelines. Furthermore, to the best of authors’ knowledge, evaluation on the long-term performance of C60/epoxy coatings with varied contents of C60 nanofillers has not been reported. 

Until recently, some research efforts have been made on corrosion protection enhancement of modified epoxy [32,33,34,35]. Li et al. [32] developed an anti-corrosion epoxy with the addition of a-zirconium phosphate nanoplatelets (ZrP). The ZrP nanoplatelets were prepared with a refluxing method. The Zirconyl chloride was refluxed in H_3_PO_4_ solution for 24 h, and the solution was then dried for another 24 h. The obtained product was grounded into fine powders. The ZrP powders were dispersed in acetone before mixing with epoxy resin. Improved corrosion resistance was confirmed by the potentiodynamic polarization and EIS measurements. Zhang et al. [33] successfully used modified silicon nitride powders to enhance the corrosion performance of epoxy coatings. The silicon nitride powders were kept in a vacuum chamber at 80 °C for 24 h before the fabrication procedure. Then the powders were dispersed into methacryloxy propyl trimethoxyl silane with ultrasonication, and deionized water, ethyl alcohol, and acetic acid were added into the solution, while the PH value was adjusted between 3.5 to 5.0. The solution was mechanically stirred for 3 h in a water bath, and the modified silicon nitride powders were obtained after the solution was dried in an oven. Stronger barrier performance was observed in the epoxy coating with the addition of silane functionalized silicon nitride. Chhetri et al. [34] developed functionalized APTES–Mo–LDH reinforced epoxy for corrosion protection application. The Mg(NO_3_)_2_·6H_2_O and Al(NO_3_)_3_·9H_2_O salt were dissolved in distilled water, and the solution were stirred with NaNO_3_ and NaOH solutions, while the PH value was maintained around 10 by adding NaOH solution. Then the obtained solution was dried inside a vacuum oven to obtained MgAl–NO_3_–LDH powder; the powder was mixed with Na_2_MoO_4_·2H_2_O with mechanical stirring for 12 h, followed by a 6 h reflux. At this stage, modified Mo–LDH was obtained, then APTES, acetic acid, and anhydrous ethanol were used to functionalize modified Mo–LDH powder into APTES-modified Mo–LDH, as named as APTES–Mo–LDH. Improved corrosion resistance and adhesion were observed in the developed APTES–Mo–LDH/Epoxy coatings. The above studies have successfully increased the corrosion protection performance of epoxy coating with their developed modification techniques; however, the applied techniques were either time-consuming or required extensive chemical solvents in the fabrication process, which showed their limitations toward large scale commercial applications.

As such, this study aimed to investigate the inclusion of fullerene-C60 in epoxy for the development of nanocomposites for oil and gas pipeline corrosion mitigation, and the nanocomposite coatings were prepared by a solvent-free facile approach. The nanoparticle size distribution was investigated using dynamic light scattering (DLS), and the dispersion was inspected by scanning electron microscopy (SEM). The mechanical and electrochemical behavior of the developed nanocomposite coatings was systemically evaluated by electrochemical impedance spectroscopy (EIS), pull-off strength test, and tensile test. In addition, the salt fog test was employed to examine the durability of the nanofiller reinforced coatings.

## 2. Experiment

This section describes the detailed experimental methods for synthesizing and characterizing fullerene-C60/epoxy coatings, including material preparation and synthesis, the dispersion method, and characterization as shown below.

### 2.1. Material

**Figure 1:**
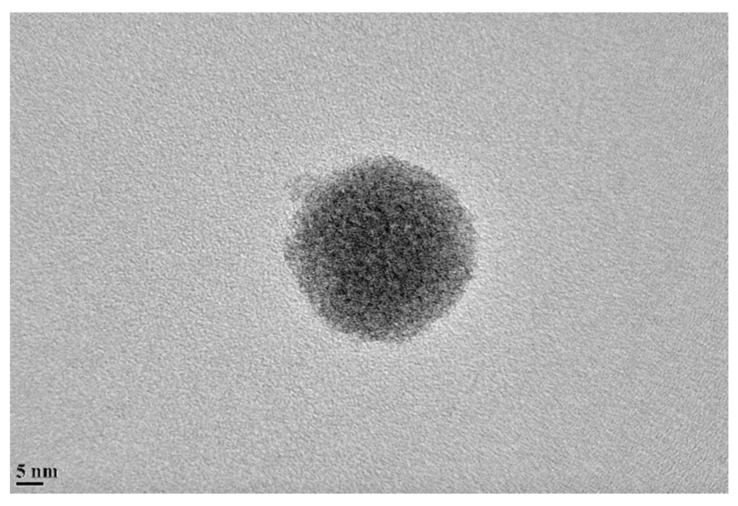
Transmission electron microscopy of a fullerene-C60 particle.

Fullerene-C60 (Sigma-Aldrich Corp., St. Louis, MO, USA) nanoparticles were purchased and used without any modification. As shown in Figure 1, the size and shape of C60 nanoparticle were examined by transmission electron microscopy (TEM), and it was clear that the as-received C60 particles were initially in agglomerates with an average diameter of 20 nm. A two-component epoxy adhesive coating was used to mix with nanofillers in this study, and the coating was based on EPON™ Resin 828 resin and Epikure 3175 (Hexion Inc., Columbus, OH, USA). The EPON™ Resin 828 resin is a pure bisphenol A/epichlorohydrin derived liquid epoxy resin and able to provide good mechanical, adhesive, dielectric and chemical resistance properties when crosslinked with EPIIKURE™ Curing Agent 3175.

### 2.2. Fabrication of Nanofiller-Reinforced Epoxy Composites and Test Sample Preparation

The dispersion of the fullerene-C60 was carried out by integration of high-speed disk (HSD) and ultrasonication, as this method can effectively disperse nanoparticles into polymer matrix. The epoxy resin was first mixed with C60 particles by using HSD dispersers (high-speed impellers) at a speed of 4000 rpm for 30 min, and the shear stress during the high-speed rotation was able to break down large aggregated particles. After the first step, the solution was subjected to ultrasonication (Misonix S1805 sonicator) with a 19-mm probe at 100% amplitude, and the process used a 30 s on/off cycle for a total duration of 60 min. After this step, a proper dispersion of C60 nanoparticles was obtained in the epoxy resin. The solution was cooled to room temperature and was then mixed with EPIIKURE™ Curing Agent 3175 with a 1:1 mole ratio at 600 rpm for another 10 min.

Nanocomposites with varied C60 weight concentration were fabricated in this study, which included 0.1, 0.5, 1.0, 1.5 and 3.0 wt.%. The developed coatings were applied on standard Q-panel steel substrates, and the commercial panels were purchased from Q-lab. Inc, with a dimension of 76 × 152 mm and a thickness of 0.8 mm. The panels were cleaned with acetone before applying the developed coatings. The coatings were then allowed to completely dry for a few days. After that, the thickness of dried films was measured, and the average thickness of 110 ± 5 μm was obtained for all the tested specimens. For simplicity, the specimens were labeled based on the weight content of nanofillers, while 0.1% F-Epoxy denotes the coating mixed with 0.1 wt.% of fullerene-C60.

### 2.3. Characterization Method

#### 2.3.1. Characterization

The characterization of nanoparticle and nanocomposite coatings was performed by several techniques, including dynamic light scattering (DLS), scanning electron microscopy (SEM) and transmission electron microscopy (TEM). A JEOL JEM-2100 (JEOL USA, Inc., Peabody, MA, USA) high-resolution analytical transmission electron microscopy (TEM) was employed to examine the shape and average size of fullerene-C60 nanoparticles (as typically shown in Figure 1). Moreover, the dispersion and stability of nanoparticles were characterized by dynamic light scattering, with a Particle Sizing Systems Nicomp 380 (PSS Nicomp, Santa Barbara, CA, USA). The average particle size distribution of fullerene-C60 was obtained for the specimens with varied weight content of nanoparticles, and the information was used to investigate the extent of agglomeration. In order to visually examine the effectiveness of employed dispersion method, scanning electron microscopy (SEM) technique was carried out to observe the distribution of nanoparticles in the epoxy matrix, with a JEOL JSM-7600F field-emission SEM (JEOL USA, Inc., Peabody, MA, USA). Meanwhile, after tension test, SEM images of fracture surface were used to analyze the impact resistance and fracture toughness for the dogbone shaped specimens.

#### 2.3.2. Corrosion Resistance of the Composite Coating Using EIS

The corrosion protection performance of the developed coatings can be effectively evaluated by the electrochemical behavior obtained from electrochemical impedance spectroscopy (EIS) test [36]. The EIS test was employed by using Gamry equipment (Reference 600 potentio/Galvanostat/ZRA); a saturated calomel electrode reference electrode was used as the reference electrode, while a platinum mesh and the test panel were worked as the counter electrode and working electrode, respectively. A glass tube with a diameter of 30-mm was clamped on the panel and filled with 1.0% of NaCl solution during the test. The measurements were collected in the frequency range of 10^5^ to 10^−2^ Hz, and the obtained data was described as impedance spectra. The impedance-frequency plot analysis was used to provide detailed information about the corrosion potential of coatings.

Salt spray test (ASTM B117) was applied as an accelerated durability test for the prepared coatings to examine their long-term performance. The specimens were exposed to salt fog spray with evaluated temperature in a Q-Fog CCT chamber (Q-Lab Corporation, Cleveland, OH, USA) for 200 h, and EIS measurements were conducted before, 100 h, and 200 h after the exposure.

**Figure 2:**
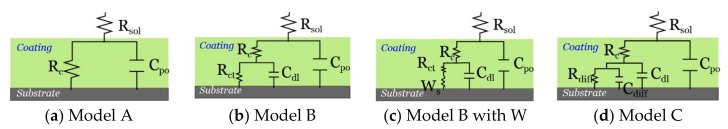
Equivalent electrical circuit models at four stages: (**a**–**d**). (**a**) Model A; (**b**) Model B; (**c**) Model B with the Warburg impedance element (W); (**d**) Model C.

For further investigation, the obtained data from EIS measurement were fitted into equivalent electrical circuit models (EEC). As described in Figure 2, the coating degradation process was characterized into four stages, and each stage could be represented by one EEC model. The EEC model consists of *R*_sol_ (solution resistance), *R*_c_ (coating resistance) and *C*_po_ (constant-phase element of the coating) is labeled as model A, indicates that coating behaves an intact layer to protect substrate. After that, once the coating was damaged and could not prevent electrodes from penetrating the coating layer to contact with substrate, that is, the corrosion reaction was initiated, then the coating reached the second stage. In this case, *R*_ct_, charge transfer resistance, and *C*_dl_, constant phase element of double-charge, were added into the EEC model, and the new model was named as model B. In the third stage, the model was described as model B with W, the included Warburg impedance element (W) indicating the diffusion effect dominated corrosion has occurred in the system. In the final stage, the coating suffered severe corrosion damage, and a thin corrosion product layer was accumulated by a large amount of corrosion products. The new parameters, including constant phase element of diffusion capacitance (*C*_diff_) and diffusion resistance (*R*_diff_) were joined to represent the corrosion product layer.

**Figure 3:**
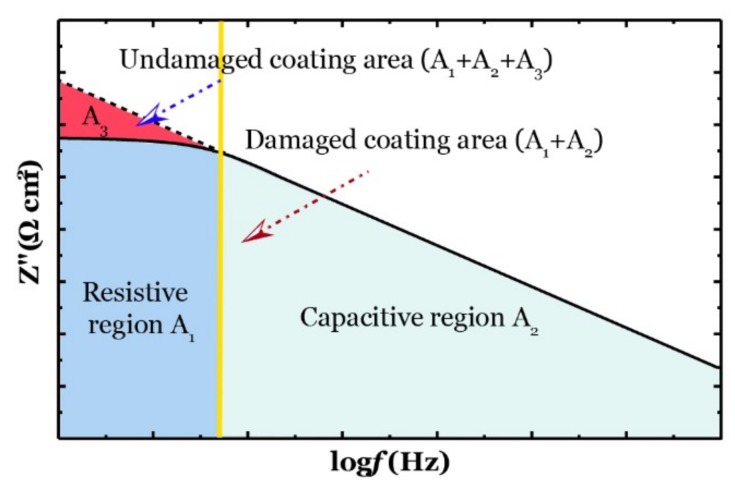
Corrosion protection index for the coating degradation assessment.

As mentioned above, the degree of coating damage over exposure time can be evaluated using EIS data. In this study, a new corrosion protection index was introduced to effectively describe the coating corrosion resistance, and the formulation was modified from previous researchers’ work [37,38]. Therefore, the coating corrosion protection index, CCPI, could be obtained through the EIS data at a wide range of frequencies, as shown below:

(1) $$\mathrm{CCPI}\left(\%\right)=\left(\frac{A_{1}+A_{2}}{A_{1}+A_{2}+A_{3}}\right)\times 100$$

where (*A*_1_ + *A*_2_) is the area under the impedance curve in log-log axes for a damaged coating, and (*A*_1_ + *A*_2_ + *A*_3_) represents the impedance plot for an ideally intact coating (see Figure 3). In this case, a larger value of *CCPI* indicated less coating damage, and when the value reached up to 100%, the coating behaved as an intact layer for corrosion protection.

#### 2.3.3. Adhesion of the Composite Coating Using Tensile Button Testing

The adhesive bonding strength between coating and substrate was evaluated by the pull-off tensile test. Before the test, dollies were glued to the surface of specimen. Then the dollies were pulled vertically away from substrate until completely detached, and the adhesion strength was measured. The test area was abraded with a 100-grit sandpaper to enhance the bonding between dollies and coating, and then the dollies were glued on the abraded surface. The samples were kept in room temperature for 24 h, which allowed the glue to dry. Before the adhesion test applied, the test area was isolated by using die cutting.

#### 2.3.4. Tensile Strength, Ultimate Strain, and Young’s Modulus

The coupon tensile test was conducted to characterize the tensile properties of nanofiller reinforced polymer composites. The test was carried out by following ASTM D638 standard with a Shimadzu’s EZ-X tester (Shimadzu Scientific Instruments, Columbia, MD, USA). Tensile strength was applied to elongate the sample with a testing speed of 1mm/min until the sample broke in the narrow test section. In this test, maximum tensile strength, strain at failure, and Young’s modulus of each specimen were calculated.

## 3. Results and Discussion

### 3.1. Particle Size Distribution and Dispersion

**Figure 4:**
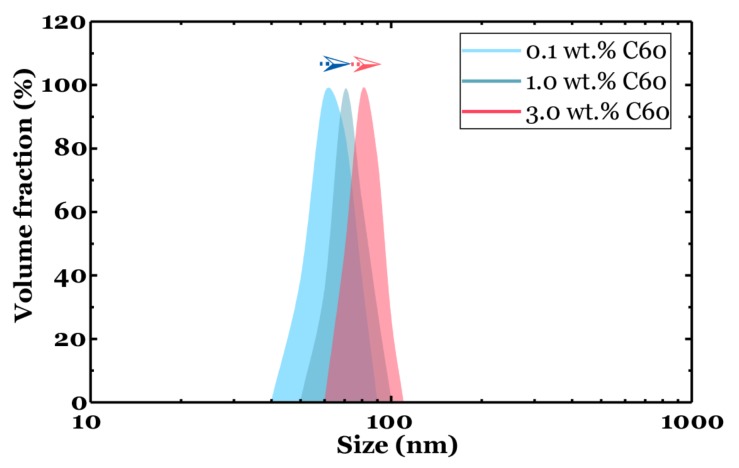
Particle size distribution of fullerene-C60 nanocomposites.

The DLS measurements confirmed that the developed dispersion method could effectively prevent the C60 particles from forming large agglomerates. The obtained results revealed that the C60 samples were dispersed into nano-sized particles with a diameter between 40 to 100 nm, and the average particle size did not significantly increase with the increasing weight concentration of nanofillers (Figure 4). A slight increase in particle size was observed, as the average particle diameter was around 65, 70, and 80 nm for the sample with 0.1, 1.0 and 3.0 wt.% C60, respectively.

**Figure 5:**
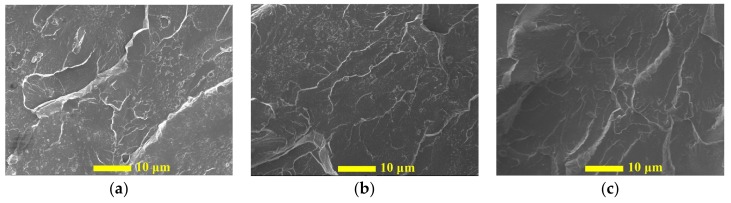
Cross-sectional SEM image of (**a**) 0.1 wt.%, (**b**) 1.0 wt.%, and (**c**) 3.0 wt.% C60/epoxy.

The cross-sectional SEM images of epoxy samples with varied content of C60 were presented in Figure 5a through to Figure 5c. The observation showed a strong agreement with results from the particle size distribution test, and the C60 particles behaved with good compatibility and a high dispersion level in the epoxy matrix, in which no large agglomerate was observed in all the tested samples. These results also indicated that fullerene particles exhibited a weak tendency to form agglomerates due to their unique spherical shape [17].

### 3.2. Barrier Performance of the New Composite Coatings

**Figure 6:**
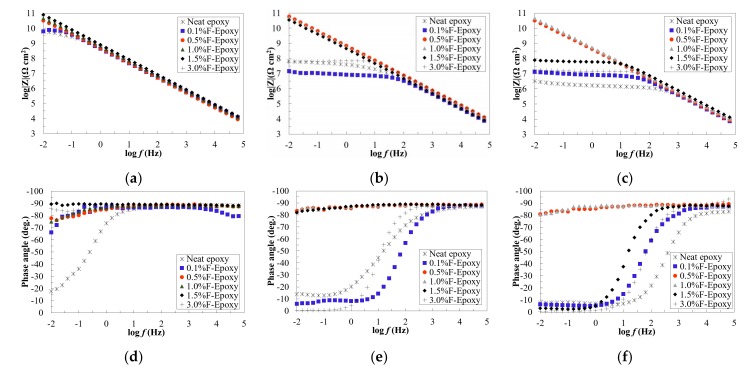

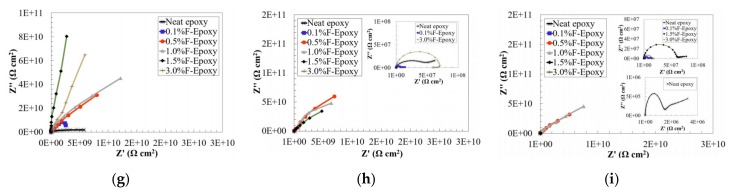
Impedance, phase angle and Nyquist plots of C60/epoxy coatings under (**a**,**d**,**g**) onset, (**b**,**e**,**h**) 100-h, and (**c**,**f**,**i**) 200-h exposure.

The corrosion barrier performance of the samples loaded with fullerene-C60 particles was evaluated by way of an electrochemical impedance spectroscopy (EIS) test. The EIS measurement was utilized before, and 100 h, and 200 h after exposure and the results were presented in terms of impedance and phase angle plots, as shown in Figure 6a–f. According to the collected data, the neat epoxy coating behaved fair in terms of corrosion protection for the substrate. However, as a clear bend was observed at the low-frequency region of impedance curve, it could be understood that the neat epoxy coating could not act as a solid barrier layer against corrosive media [39]. Researchers have suggested that micro-pores would be generated into a neat epoxy coating during the curing process, and these voids allowed corrosive media to penetrate through the coating and initiate a corrosion reaction at substrate, as confirmed elsewhere [40,41]. This was further confirmed by data in Table 1, where the neat epoxy coating behaved at the third stage of corrosion process, and the Warburg impedance element (W) indicates that the corrosion was diffusion dominant. As shown in Figure 6b,c, a clear degradation was observed in the neat epoxy coating during the exposure, and the degradation level was developed over time, suggesting that the coating failed to provide long-term corrosion protection for metallic substrate.

Obviously, higher content fullerene-C60 particles (from 0.5 to 3.0 wt.%) provided stronger corrosion resistance than the neat epoxy. Overall, it turned out that model A was suitable for the groups with 0.5 to 3.0 wt.% fullerene-C6o particles, which meant that the coatings were providing perfect corrosion resistance (Table 1). As shown in Figure 6a, similar to the neat epoxy, the group with 0.1 wt.% of fullerene-C60 exhibited low corrosion resistance and a clear bend was observed in the phase angle curve in the tested frequency region. The results suggested that there was no noticeable reinforcement on corrosion resistance when low content (0.1 wt.%) of fullerene-C60 was incorporated into epoxy resin. Differently, all the other fullerene-C60/epoxy groups exhibited higher impedance, suggesting improved corrosion barrier performance was obtained by the addition of C60 nanofillers.

After 200 h of exposure, similar to the fresh stage, the results from 0.5% and 1.0% F-Epoxy group showed their extraordinary anti-corrosion performance, regardless of exposure time. High impedance value was maintained during exposure, as the impedance value was over 10^10^ Ω/cm^2^ during the exposure. Additionally, the 0.5% and 1.0% F-Epoxy groups remained in model A during the whole exposure, which confirmed that 0.5 and 1.0 wt.% of fullerene-C60 could dramatically improve the corrosion resistance of epoxy coatings. The 0.1%F-Epoxy exhibited the lowest corrosion resistance, and this result confirmed the conclusion above which low content (0.1 wt.%) of fullerene-C60 would not improve the corrosion resistance of epoxy. Additionally, it was clear to observe that the 3.0% F-Epoxy coating started to delaminate and damage due to the salt spray. This phenomenon indicated that the high content of fullerene-C60 particles (3.0 wt.%) provided a short-term reinforcement on corrosion resistance, but it was weak in durability for a long-term test.

**Figure 7:**
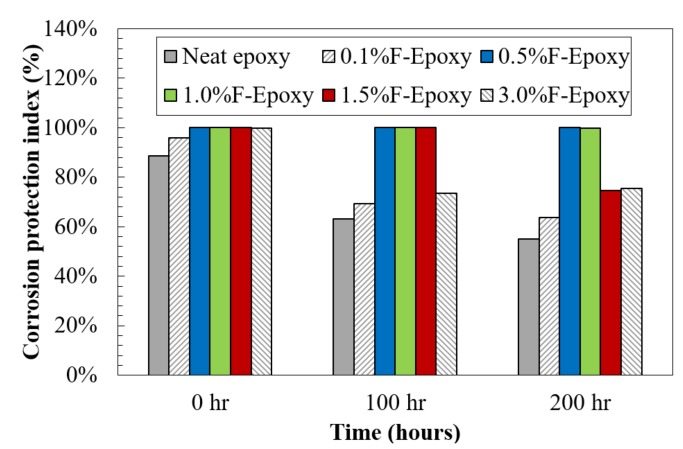
Coating corrosion protection index of C60/epoxy coatings.

Figure 7 was plotted for the results of coating corrosion protection index. Clearly, the results were in strong agreement with the previous observation from Bode plots. The neat epoxy demonstrated the weakest anti-corrosion performance, with significant development of coating damages, as compared to all the nanofiller/epoxy coatings during the exposure. The degree of coating damage at the initial moment and after exposure was reduced by the addition of C60. The samples that were reinforced by 0.5 and 1.0 wt.% C60 nanoparticles showed the highest corrosion protection and more stability against the severe environment. During the exposure, 0.5 and 1.0 wt.% C60-Epoxy remained 100% undamaged, indicating that the coating system was acting as an intact layer against the penetration of corrosive media.

### 3.3. Adhesive Bond Strength of Nano-Reinforced Composites to the Substrate

Understanding the bonding properties between the protective coating and the substrate could assist to characterize corrosion protection properties. The pull-off strength (adhesion) was measured by following ASTM D4541 to evaluate the tensile bond strength of nano-reinforced epoxy coatings with varied C60 concentrations. The performance of the neat epoxy was employed as a reference, and the adhesive strength was close to 3.05 MPa.

**Figure 8:**
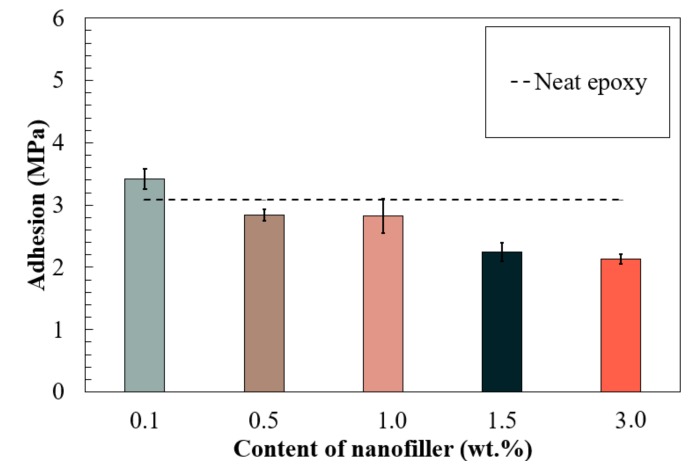
Pull-off strength of the nanocomposites with varied types of carbon nanofillers.

The pull-off bond strength over the nanofiller concentration is illustrated in Figure 8. Different to the corrosion barrier performance, the adhesive strength increased in the coating with 0.1 wt.% of fullerene-C60 and at maximum strength, reached 3.42 MPa. With 0.5 and 1.0 wt.% C60 particles, the adhesion strength reduced to 2.84 MPa but remained close to neat epoxy. Adhesion decreased when the coatings had a higher content of fullerene-C60 (1.5 to 3.0 wt.%). For instance, the adhesion dropped to 2.14 MPa in the sample with 3.0 wt.% C60 particles.

**Figure 9:**
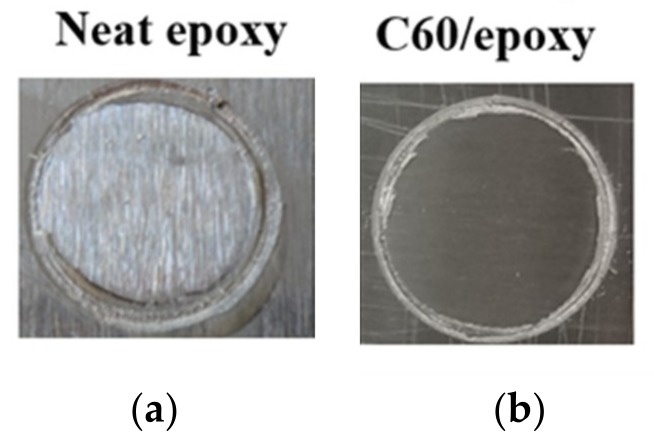
The adhesion failure mode of (**a**) neat epoxy and (**b**) epoxy with 1.0 wt.% C60 nanofillers.

Different interfacial failure modes could reveal the level of bond strength between coating and substrate [42]. The failure mode of the coatings with and without C60 particles after the pull-off strength test as illustrated in Figure 9 demonstrated that the coatings were wholly detached from the substrate for both neat epoxy and C60/epoxy groups, indicating that an adhesive failure mode was obtained, as adhesive strength was less than their tensile strengths [43].

### 3.4. Tensile Behavior of the Nanocomposite Coating

Tensile properties of the nanofiller reinforced epoxy account for whether the developed coating could provide high damage tolerance for real-world applications. The analysis of tensile properties of nanofiller reinforced epoxy was performed by a tensile test, following ASTM D638. The nanocomposites were evaluated by measuring the maximum tensile stress, strain at failure, and the Young’s modulus during the test. 

**Figure 10:**
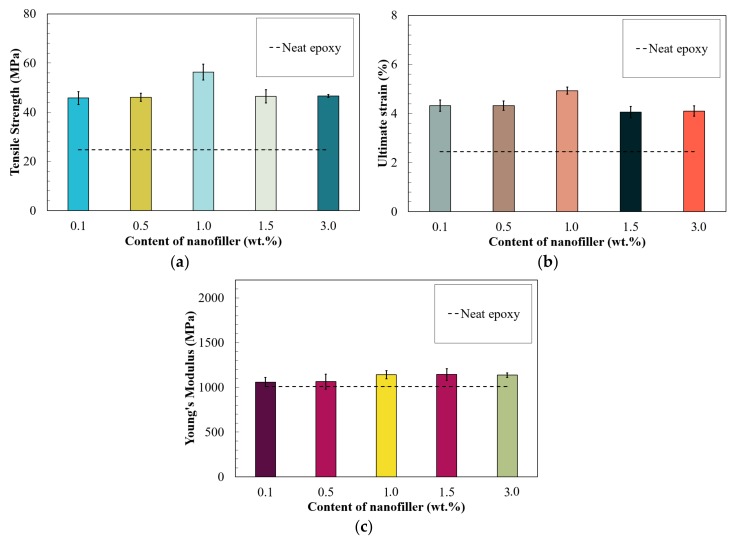
(**a**) Tensile strength, (**b**) ultimate strain, and (**c**) Young’s modulus of nano filler/epoxy composites.

Figure 10a shows the maximum tensile stress of the tested nanocomposites at varied concentrations. The results indicated the C60 had a dramatic reinforcement in tensile strength; hence; the tensile stress of all the tested fullerene-C60/epoxy groups were higher than 45 MPa while of neat epoxy it was 24 MPa. Additionally, a gradual increase in the tensile stress was observed in 0.1 to 1.0 wt.% fullerene-C60 groups. The maximum stress was 56 MPa and found in the 1.0%C60-Epoxy, increased 130% compared to that of the pure epoxy group. Material degradation was found in the group with higher concentration (3.0 wt.%) of C60 nanofillers.

Furthermore, a similar tendency was observed in ultimate strains presented in Figure 10b. The ultimate strain of all the tested nanocomposites was increased by the addition of fullerene-C60 particles; thus, the ultimate strains of all F-Epoxy groups were larger than 4.0% while neat epoxy was 2.4%. The greatest improvement of ultimate strain was obtained by the addition of 1.0 wt.% of fullerene-C60, and the ultimate strain was 4.9%, which was as twice as neat epoxy samples.

The Young’s modulus of the nanocomposites exhibited an identical trend as observed in their tensile strength and strain (Figure 10c). The value of Young’s modulus for the fullerene-C60/epoxy groups was increased in all the tested nanocomposites. Similar to tensile strength and strain, the value of Young’s modulus values was increased from 0.1 to 1.0 wt.% of fullerene-C60 groups. However, unlike the other properties, no degradation was observed in the nanocomposites with a higher amount of fullerene-C60 (1.0 to 3.0 wt.%).

**Figure 11:**
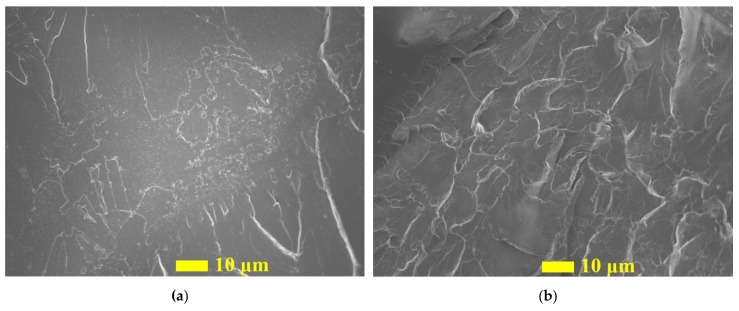
The fracture surface of (**a**) neat epoxy, (**b**) C60/epoxy with 1.0 wt.% of C60.

The fracture surfaces for specimens fractured under tensile stress are shown in Figure 11. The relatively smooth surface was observed in pure epoxy comparing with nano-reinforced composites. This typical brittle fracture from pure epoxy represented the low impact resistance and fracture toughness of the non-reinforced composite. In contrast, the fracture surface was significantly rougher for the composite containing C60 than the pure epoxy (Figure 11b), where higher surface roughness and more compacted cleavages were observed, indicating signs of higher energy absorption and better fracture resistance. This observation is consistent with the experiment results that higher strain at failure was obtained from C60 reinforced epoxy.

## 4. Conclusions

Nano reinforced composites were fabricated by incorporating fullerene-C60 into the epoxy matrix, potentially for high-performance coatings in pipelines. Highly dispersed nanoparticles were achieved using a solvent-free dispersion method through the integration of ultrasonication and high-speed disperser. The level of particle dispersion was examined via DLS measurements and cross-sectional SEM images. Major properties affecting the performance of corrosion protection coatings, including corrosion barrier property, mechanical strength, and long-term durability, were systemically characterized and the findings are summarized as follows:

(a) The fullerene-C60/epoxy coatings exhibited improved electrochemical, mechanical properties with excellent durability, indicating the coatings enabled protection of the substrate against a harsh environment with corrosive media that oil/gas pipelines often experience.
(b) Particle distribution results from DLS measurements revealed the developed dispersion method effectively overcome agglomeration, and no large particles were observed in all the tested samples.
(c) The incorporation of fullerene-C60 as a coating additive led to dramatically improved corrosion resistance, as suggested by EIS results. Excellent barrier performance was observed in the samples with higher content fullerene-C60 particles (from 0.5 to 3.0 wt.%).
(d) EIS results after salt fog exposure confirmed that nanofiller coatings could provide a much longer life as compared with the neat epoxy. Particularly, as compared to an over 50% reduction in the control samples, the coatings with 0.5 and 1.0 wt.% of fullerene-C60 particles remained intact even after 200-h exposure to salt spray, as identified on their impedance values in Bode plots.
(e) Enhancement in mechanical properties was observed in all the coatings with fullerene-C60 particles. The 1.0% F-Epoxy group exhibited the highest increase in tensile properties, including increased strength, strain, and Young’s modulus. In addition, improvement on adhesion was observed in the coating with low content of fullerene-C60 particles (0.1 wt.%).

## Figures and Tables

**Table 1:** Electrochemical impedance spectroscopy (EIS) data associated with different stages of the equivalent electrical circuit models.

Label	Content of C60 (wt.%)	Exposure to Accelerated Environmental Stresses					
		Onset		100-h		200-h	
		Electrical Circuit Models (EEC)	|Zmod|_0.01HZ_	EEC	|Zmod|_0.01HZ_	EEC	|Zmod|_0.01HZ_
Neat epoxy	/	Model B with W	6.10 × 10^9^	Model B with W	6.46 × 10^7^	Model B with W	3.29 × 10^6^
0.1%F-Epoxy	0.1	Model B with W	6.39 × 10^9^	Model B with W	1.45 × 10^7^	Model B with W	1.31 × 10^7^
0.5%F-Epoxy	0.5	Model A	3.21 × 10^10^	Model A	6.03 × 10^10^	Model A	3.22 × 10^10^
1.0%F-Epoxy	1.0	Model A	4.69 × 10^10^	Model A	4.87 × 10^10^	Model A	4.64 × 10^10^
1.5%F-Epoxy	1.5	Model A	8.04 × 10^10^	Model A	3.47 × 10^10^	Model B	7.86 × 10^7^
3.0%F-Epoxy	3.0	Model A	6.51 × 10^10^	Model B	5.85 × 10^7^	Model B	1.83 × 10^7^
